# Heavy mineral variations in mid‐Carboniferous deltaic sandstones: Records of a pre‐depositional sediment history?

**DOI:** 10.1002/dep2.128

**Published:** 2020-12-12

**Authors:** Martin Nauton‐Fourteu, Shane Tyrrell, Andrew Morton

**Affiliations:** ^1^ Earth and Ocean Sciences Sediment Origins Research Team (SORT) and Irish Centre for Research in Applied Geosciences (iCRAG) National University of Ireland Galway Ireland; ^2^ HM Research Associates St Ishmaels Haverfordwest UK; ^3^ CASP Cambridge UK

**Keywords:** Apatite–tourmaline index, Clare Basin, heavy minerals, intermediate storage, provenance, weathering

## Abstract

Sandstone composition is influenced by multiple factors, including acidic weathering, occurring during storage in the hinterland, prior to deposition. This study aims to better understand and constrain how the nature and duration of such pre‐depositional factors might impact the final sediment composition. Mid‐Carboniferous deltaic sandstones from the Clare Basin, western Ireland, for which depositional environments and provenance are well constrained, are the target of this study. Conventional heavy mineral analysis and specific heavy mineral ratios, such as the apatite–tourmaline index are utilised to examine these phenomena. Relatively high apatite–tourmaline index values observed in channelised sandstones contrast with lower values seen in sandstones associated with mouth bar and interdistributary bay facies. These variations are not linked to changes in provenance and thus potentially indicate differences in weathering intensity due to variable duration of alluvial storage. These changes are probably linked with shorter hinterland residence time in the channelised than in mouth bar and interdistributary bay sandstones. Variations are seen in the rutile‐zircon index without any clear link with facies. These fluctuations could be ascribed to variable supply from a source, which is relatively rich in rutile but poor in zircon and apatite. Despite the apatite component in these sandstones being partially derived from recycled sources, the apatite–tourmaline index stills appears to hold information on the last sedimentary cycle.

## Graphical abstract

Heavy mineral indices, and more specifically the apatite–tourmaline index (ATi), informs on pre‐depositional sediment history of Carboniferous deltaic sandstones. ATi variations correlate with changes in deltaic facies and indicate differences in weathering intensity due to variable duration of alluvial storage.

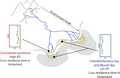

## 1 INTRODUCTION

**Figure 1:**
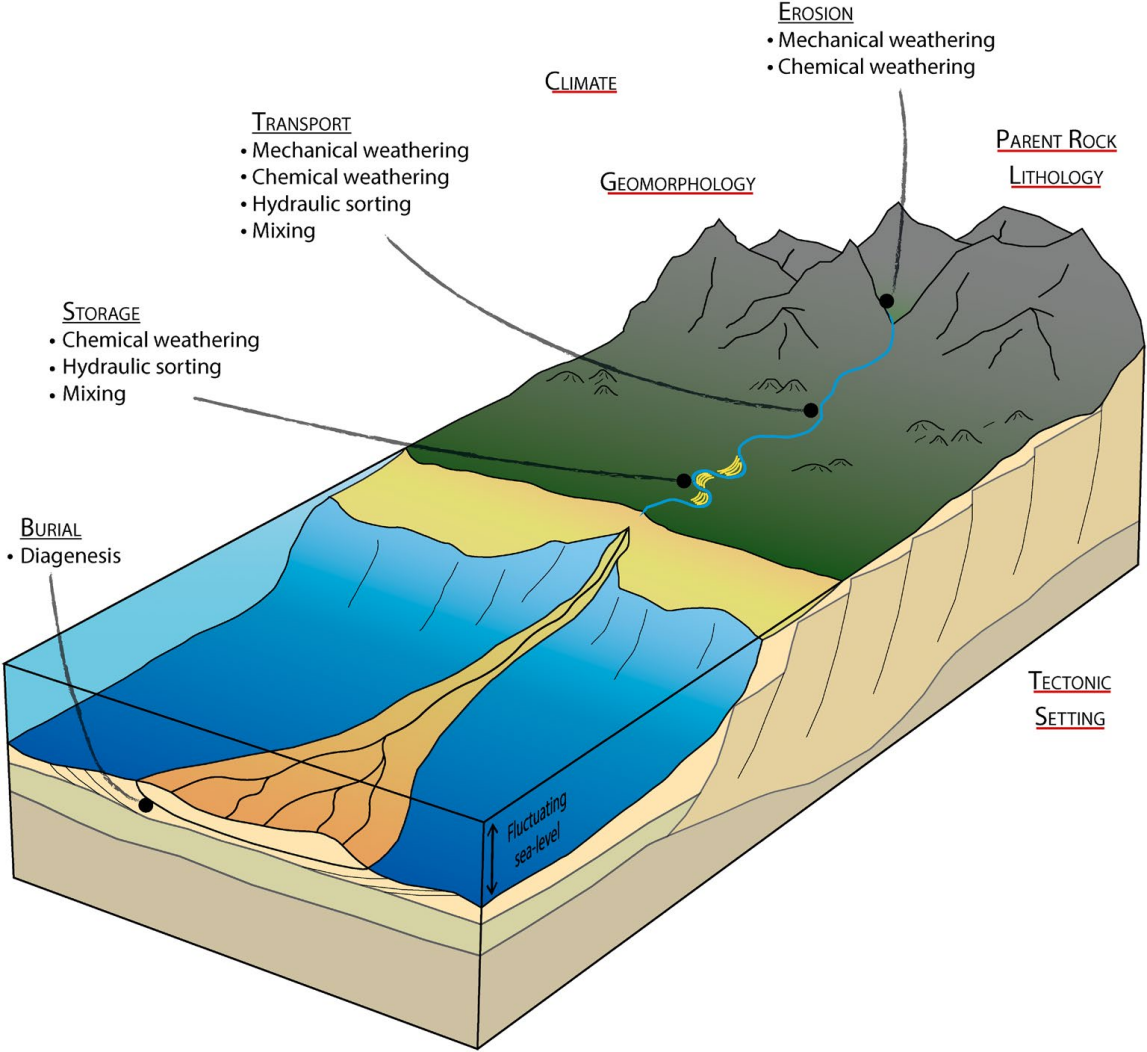
Schematic representation of the processes modifying the sediment composition during erosion, transport, intermediate storage and burial. Processes are taken from Morton and Hallsworth (1999)

From source rock to final deposition, the sediment composition is modified by various processes, occurring at different stages of the sedimentary cycle (Figure 1; Morton & Hallsworth, 1999; Weltje & von Eynatten, 2004). An improved understanding of such modifications, if identified in the final rock or sediment composition, might allow the pre‐depositional sediment history of the detrital material to be better constrained. From an applied perspective, this could help better predict reservoir quality, but could also have significant implications for palaeoweathering and palaeoclimate reconstructions (Arribas et al., 2014; Dinis et al., 2017; 2020; Tobin & Schwarzer, 2014).

Chemical weathering preferentially alters and ultimately removes grains from the sediment through either replacement by secondary minerals (e.g. clay) or by complete dissolution (Dinis et al., 2017; Garzanti et al., 2019; Johnsson & Meade, 1990). It can have an important impact on sediment composition and can sometimes be intense enough to overprint the tectonic signal, as seen in first‐cycle quartz arenites from the Orinoco drainage basin (Johnsson et al., 1988; Savage et al., 1988). Temperature, climate and groundwater pH are the key controls on chemical weathering and it is recognised that a higher degree of chemical weathering will occur in a warm and humid climate rather than in an arid environment (Chew et al., 2020; Dinis et al., 2020). For these reasons, quantifying the degree of weathering through changes in the sediment composition could potentially infer on sediment pre‐depositional history, palaeoweathering and past climate conditions (Dinis et al., 2017; 2020). Such studies could be carried out at deep geological time scales (hundreds of Myr), where the sedimentary record is preserved (Romans et al., 2016).

Chemical weathering experienced by the detritus prior to ultimate deposition, and the associated implications for palaeoclimate reconstruction, has been investigated through multiple geochemical proxies based on rock composition, especially for fine‐grained sediments and their clay fractions (Dinis et al., 2020). These proxies use the molar proportions of silicate‐bound major elements, with most of the indices comparing proportions of non‐mobile elements versus mobile elements comprised in feldspar and leached out by chemical weathering (Dinis et al., 2020; Nesbitt & Young, 1982). In sediments derived from source rocks depleted in feldspars, and in sedimentary rocks for which feldspars have been removed during burial diagenesis, these bulk rock geochemical proxies would potentially fail in identifying the extent of palaeoweathering.

The degree of chemical weathering of sediments is intrinsically linked with the time of exposure to surface or near‐surface weathering conditions. Sediment residence time in the hinterland could range from a few hours during an extreme flooding event to thousands of years, or even more in the case of alluvial storage (Lang et al., 2018). Longer exposure to the agents of chemical weathering have been observed in long‐lived river systems and during alluvial storage phases (Johnsson & Meade, 1990; Johnsson et al., 1988). Such stored alluvial sediments can then be remobilised during a river channel migration (avulsion) or eroded during a base‐level drop/lowstand, and then redeposited further down‐stream (Johnsson & Meade, 1990).

Heavy minerals (grain density >2.82 g/cm^3^) are a minor component of sandstones as they usually represent *ca* 1% of the grains in the sample (Mange & Wright, 2007). Their responses to physical and chemical weathering as well as the effects of burial diagenesis are well constrained (Morton & Hallsworth, 1999; 2007; Turner & Morton, 2007). The use of pairs of heavy minerals has proven useful in investigating specific processes which have the potential to modify the sediment composition. For example, apatite and tourmaline are equally impacted by hydraulic processes such as sorting or mixing (Morton & Hallsworth, 1999). However, they show different stabilities regarding chemical weathering, with apatite more likely to be weathered in acidic conditions than ultra‐stable tourmaline (Morton & Hallsworth, 1999). Both minerals have been recognised as being negligibly impacted by burial diagenesis (Morton & Hallsworth, 2007; Turner & Morton, 2007). Variations in the relative abundance of these minerals, through the apatite–tourmaline index (ATi), has been used to investigate pre‐depositional modification of the sediment. It has been shown that weathering during alluvial storage has an impact on ATi values, with lower values observed after a storage phase or longer sediment floodplain residence time (Hurst & Morton, 2014; Morton & Johnsson, 1993; Morton et al., 2012). While in arid and semi‐arid depositional environments, variations in ATi would more likely be an indicator of changes in provenance, in fluvio‐deltaic sandstones deposited under humid‐tropical settings, the ATi is likely to be strongly influenced by weathering during alluvial storage (Morton & Johnsson, 1993). In contrast to ATi, the rutile–zircon index (RuZi) and the chrome spinel–zircon index (CZi) are considered to be more diagnostic of changes in provenance and source rock lithology (Morton & Hallsworth, 1999).

This study aims to utilise heavy mineral analysis to better understand modifications to sediment composition which can occur in the hinterland, prior to deposition, and more specifically better constrain the impacts of sediment hinterland residence time and associated palaeoweathering. The Tullig Cyclothem of the mid‐Carboniferous Clare Basin, western Ireland, for which provenance has been previously constrained, is used as a case study (Nauton‐Fourteu et al., 2020). Such sedimentary rocks were deposited as a response to the high‐frequency sea‐level fluctuation intrinsically linked with climate, thus potentially recording variations of palaeoweathering conditions in the hinterland (Pyles, 2008). Near laterally continuous, coastal outcrops and sedimentary successions, representing various facies associated with deltaic environments, from the study area allow for a high‐resolution heavy mineral analysis to be performed and should hold information on the sediment pre‐depositional history. Deltaic, and associated sub‐environments, are an excellent target for such an investigation as they comprise a range of depositional energies influenced by sea‐level fluctuations.

## 2 GEOLOGICAL BACKGROUND

### 2.1 Geological setting

The Clare Basin is a Serpukhovian–Bashkirian (*ca* 331–315 Ma) siliciclastic infill from deep‐water to shallow marine settings. The sedimentary successions observed on coastal outcrops in both County Clare and County Kerry have been extensively studied over the past decades (Collinson et al., 1991; Pierce et al., 2017; Pyles, 2008), and generate interest as deep water and shallow marine reservoir analogues (Martinsen et al., 2017; Pyles, 2008; Stirling, 2003). The Clare Basin is located above the Iapetus Suture, which represents the collision between Avalonia‐Baltica and Laurentia during the late Silurian (Fairey et al., 2018; Waldron et al., 2014). The subsidence of the basin is generally believed to be linked with the reactivation of Iapetus Suture structures; however, there are contrasting models to explain how this occurred (Martinsen et al., 2017; Nauton‐Fourteu et al., 2020; Pyles, 2008; Wignall & Best, 2016).

**Figure 2:**
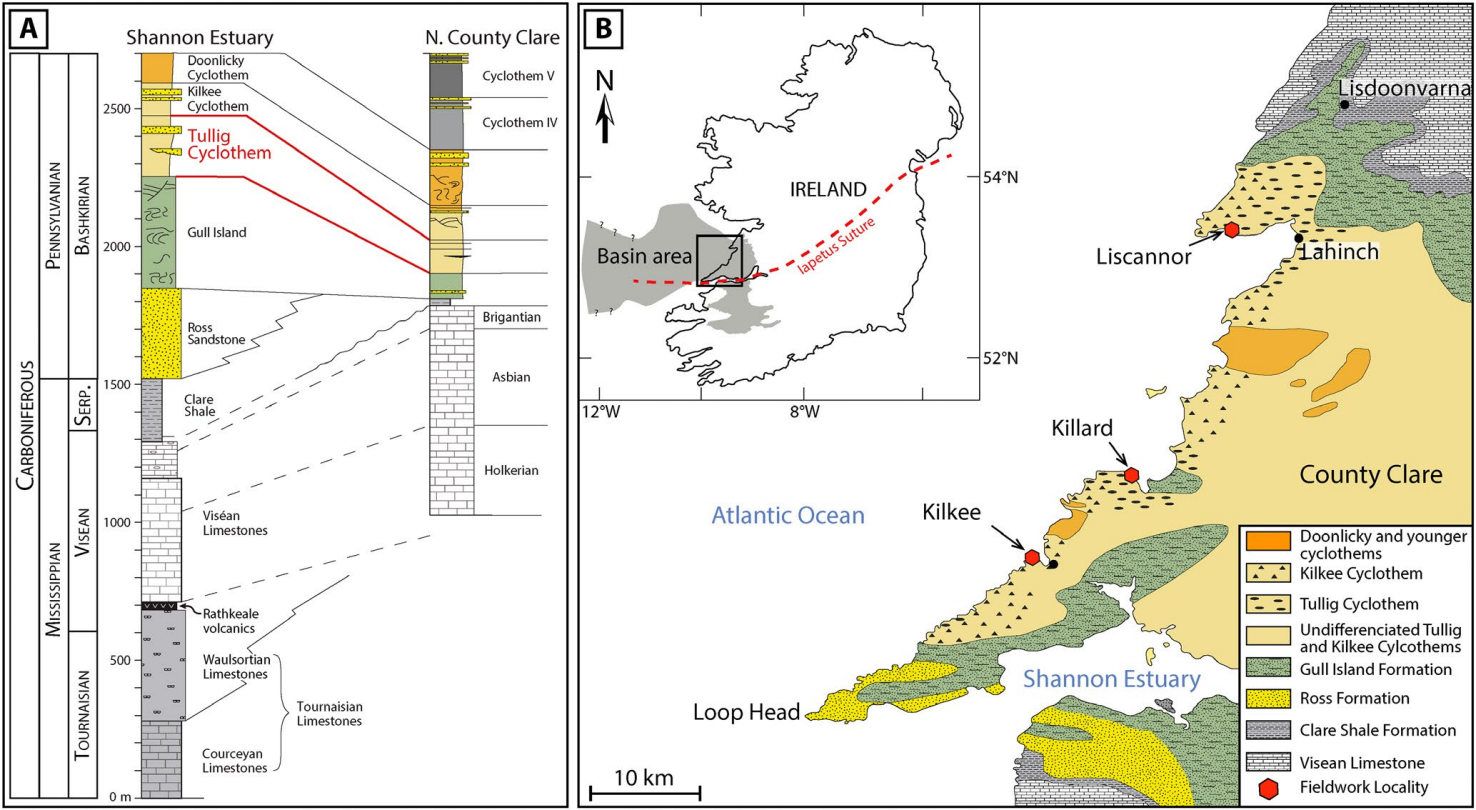
(A) Stratigraphic representation of the sedimentary succession in the Clare Basin (modified from Best & Wignall, 2016). (B) Geological map of the Clare Basin with the location of the three studied outcrops (modified from Best & Wignall, 2016 and Pointon et al., 2012). The basin area is from Geological Survey Ireland (GSI) Bedrock geology 1:1 million and Croker (1995)

In the early Carboniferous, Ireland was experiencing a humid‐tropical, non‐seasonal climate, near the equator (Elliott et al., 2000). A global climate transition from greenhouse to icehouse occurred resulting in multiple phases of glaciation and deglaciation and high‐frequency (*ca* 65 kyr)—high‐amplitude (60 ± 15 m) forced eustatic changes in sea level (Davies et al., 2012; Davydov et al., 2012; George, 2000; Pyles, 2008). The stratigraphy of the Clare Basin, constrained from goniatite‐bearing marine bands, has been divided into two groups: the Shannon Group and the Central Clare Group (Collinson et al., 1991; Rider, 1974; Figure 2A). The Shannon Group corresponds to the deep water and early fill of the basin whereas the Central Clare Group contains five deltaic cyclothems (Rider, 1974). The deposition of the five cyclothems was controlled by sea‐level change, with deposition of coarsening upwards packages during sea‐level fall, and marine bands representing maximum flooding surfaces in between each cyclothem (Collinson et al., 1991; Pulham, 1989; Rider, 1974).

The Tullig Cyclothem is composed of three types of sequence: (a) 26–120 m thick coarsening upward sequences representing the delta front; (b) 2.5–14 m thick coarsening upward sequences interpreted as interdistributary bay deposits; and (c) up to 70 m erosive channel sand body sequences (Pulham, 1989). The Tullig Sandstone Member consists of an erosively based sand body comprising several stacked individual channels up to 35 m thick to the south of the basin (Shannon Estuary), and single channels up to 15 m thick to the north (Liscannor; Pulham, 1989). The Tullig Sandstone is interpreted either as a deltaic channel sandstone (Pulham, 1989; Rider, 1974) or sand filling incised valleys created during a relative fall in sea level (Hampson et al., 1997). The transition from the Tullig to the Kilkee Cyclothem is marked by a distinct marine band containing the goniatite *Reticuloceras stubblefieldi* (Collinson et al., 1991; Rider, 1974). To the south of the basin, the Moore Bay Sandstone is a sand body *ca* 30 m thick stratigraphically located above the Tullig Sandstone and below the marine band transitioning to the Kilkee Cyclothem (Wignall & Best, 2000).

Sandstones from the Tullig Cyclothem of the Clare Basin have been classified as fine to medium quartz arenites, with finer grain sizes observed in mouth bar and interdistributary bay sandstones than in channelised sandstones (Nauton‐Fourteu et al., 2020). Significant quartz overgrowths are common, resulting in an intra‐grain porosity close to zero, highlighting the potential significant impact of burial diagenesis.

### 2.2 Provenance of the Tullig Cyclothem

Sedimentary routing of sandstones from the Tullig Cyclothem has been debated over the past decades (Collinson et al., 1991; Martinsen & Collinson, 2002; Pulham, 1989; Rider, 1974; Wignall & Best, 2000; 2002; 2016). Two main contrasting sedimentary routing models were suggested, based on the basin stratigraphy, palaeocurrent indicators and basin geometry. The first one involves sourcing from the north‐west and the Tullig Cyclothem prograding towards the south‐east (Collinson et al., 1991; Elliott et al., 2000; Martinsen & Collinson, 2002; Pulham, 1989; Pyles, 2008). The second one differs with sourcing from the south‐west and a delta prograding towards the north‐east (Wignall & Best, 2000; 2002; 2016).

A multi‐proxy provenance study of the Tullig Cyclothem has allowed for detailed sources and sediment routing interpretations (Nauton‐Fourteu et al., 2020). The most recent provenance model suggests that sandstones of the Tullig Cyclothem are derived from multiple sources from the south and south‐west, with fresh input from peri‐Gondwanan terranes and recycling of Laurentian and Caledonian material through Devonian sedimentary rocks. This previous study ascribes the compositional maturity of these sandstones to sedimentary recycling rather than the chemical weathering that could have occurred during transport (Nauton‐Fourteu et al., 2020). Apatite U‐Pb geochronology coupled with trace element data suggest that Caledonian (potentially Donegal and Galway granites) apatite has survived recycling through the Old Red Sandstones of the Dingle Basin (Nauton‐Fourteu et al., 2020).

## 3 METHODOLOGY

Twenty one sandstone samples were collected from three locations along the coast of County Clare, near Liscannor (52°56′14.40″N; 9°25′45.33″W), near Killard (52°45′4.69′N; 9°32′53.22′W) and near Kilkee (52°40′49.46″N; 9°40′35.21″W; Figure 2B). Sections were logged and sandstones sampled targeting the different deltaic sequences detailed in Pulham (1989) and Nauton‐Fourteu et al. (2020). To minimise the potential impact of weathering at the outcrop, only fresh samples were collected.

Samples were crushed using a jaw crusher. The 63–125 µm fraction was separated and processed through heavy liquid separation (bromoform, 2.8 g/cm^3^). This specific choice of grain size to be analysed is based on Morton (2012). The heavy mineral fraction was mounted in Canada Balsam and grains were identified using their optical properties under a polarizing microscope as in Mange and Maurer (1992). In order to obtain heavy mineral assemblages, counting using the ribbon method was achieved on a minimum target of 200 non‐opaque grains (Morton et al., 2013). For minerals involved in the determination of indices (apatite, tourmaline, rutile, zircon and chrome spinel), the target was to obtain a minimum of 200 grain counts per index. Some samples with low heavy mineral yield did not allow the desired 200 counts to be reached (Table S1). Four samples with heavy mineral separates containing mainly micas, carbonates, chlorite and opaque grains could not be used for interpretation. Heavy mineral indices were calculated following Morton and Hallsworth (1999) where ATi = (apatite count/total apatite plus tourmaline) × 100; RuZi = (rutile count/total rutile plus zircon) × 100 and CZi = (chrome spinel count/total chrome spinel plus zircon) × 100. The indicator of mineralogical maturity zircon–tourmaline–rutile index (ZTR) is calculated as the sum of ZTR grain counts over the total of transparent heavy minerals (Hubert, 1962).

## 4 RESULTS

**Figure 3:**
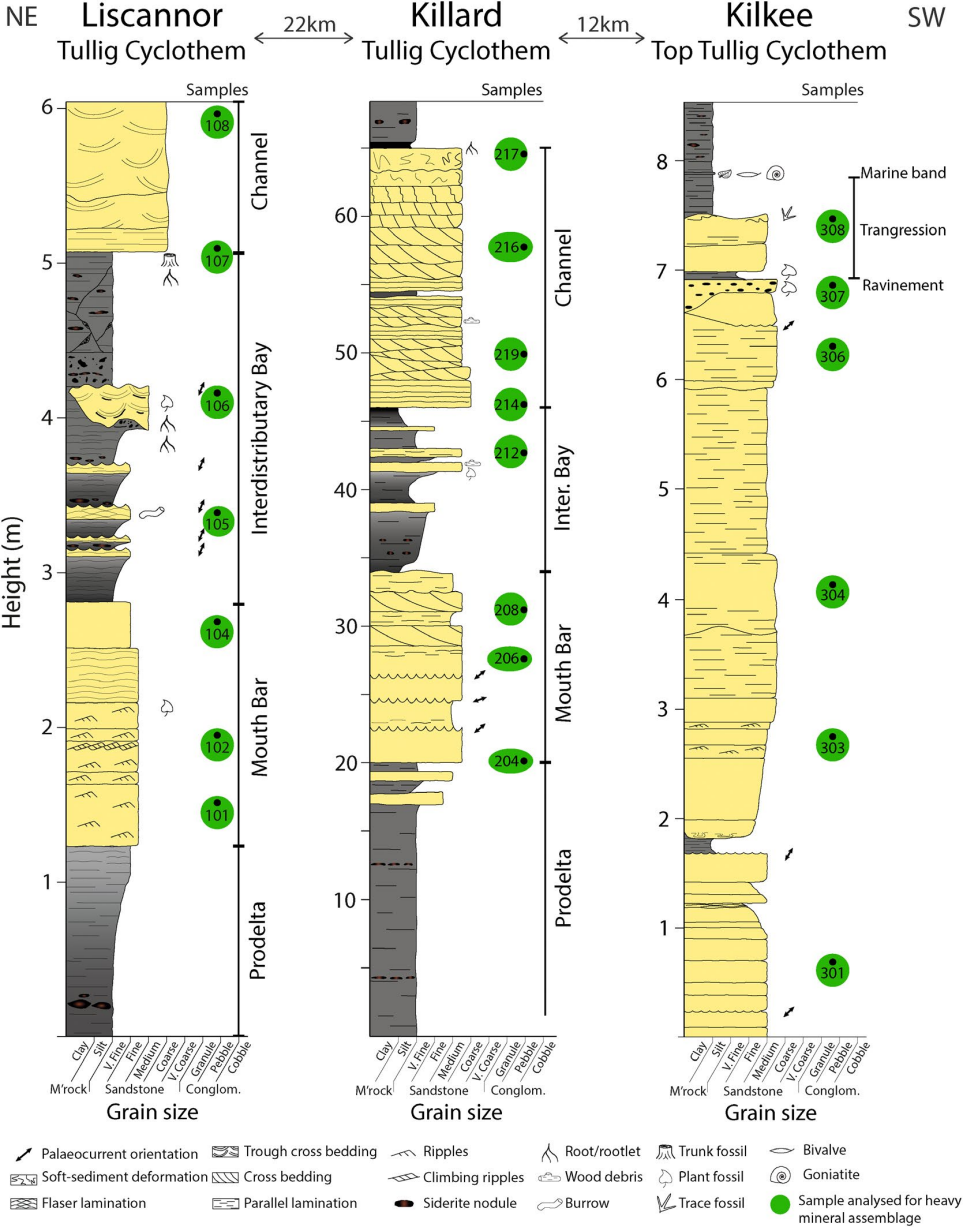
Stratigraphic logs representing the sedimentary successions at Liscannor, Killard and Kilkee, with sample locations. Modified from Nauton‐Fourteu et al. (2020)

Tullig Cyclothem sedimentology at the three locations has been previously investigated and interpreted (Best et al., 2016; Blanchard et al., 2019; Nauton‐Fourteu et al., 2020). Both Liscannor and Killard outcrops display a delta progradation with prodelta, mouth bar, interdistributary bay facies and the channelised Tullig Sandstone (Pulham, 1989; Figure 3). These facies have been discussed in detail in Nauton‐Fourteu et al. (2020). At Kilkee, the logged section displays the Moore Bay Sandstone, the top part of the Tullig Cyclothem, which consists of a shelf sand body reworked by waves during a sea‐level rise (Best et al., 2016; Blanchard et al., 2019; Nauton‐Fourteu et al., 2020; Figure 3).

**Figure 4:**
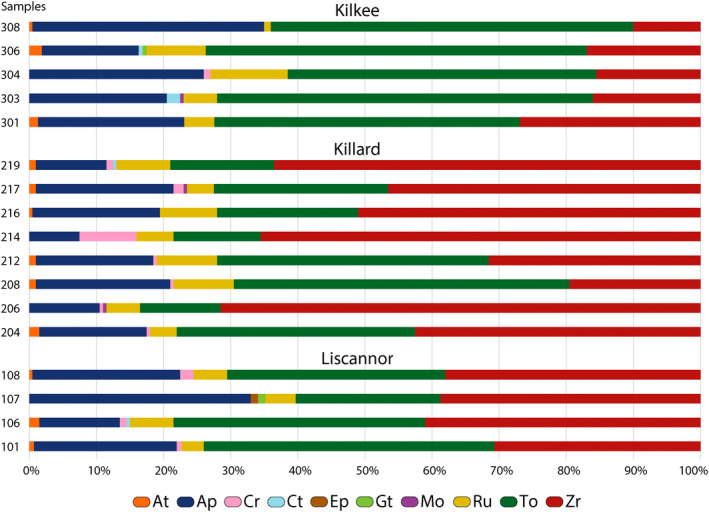
Heavy mineral assemblage results from the three studied localities. At: anatase, Ap: apatite, Cr: chrome spinel, Ct: chloritoid, Ep: epidote, Gt: garnet, Mo: monazite, Ru: rutile, To: tourmaline, Zr: zircon

Heavy mineral assemblages are represented in Figure 4, with data available in Table S1. Zircon and tourmaline dominate the assemblage and ZTR indices are relatively high, with values ranging from 64.8% to 88.5%. The amount of chrome spinel is relatively low, with values of *ca* 0.5% except for sample 214 in Killard (8.5%). Garnet, epidote and chloritoid are either absent or present in low amounts. Apatite is observed in all the samples with abundances ranging from 7.5% to 34.5% of the heavy mineral total. Samples from Kilkee seem to contain proportionally more tourmaline and less zircon than samples from Liscannor and Killard.

**Figure 5:**
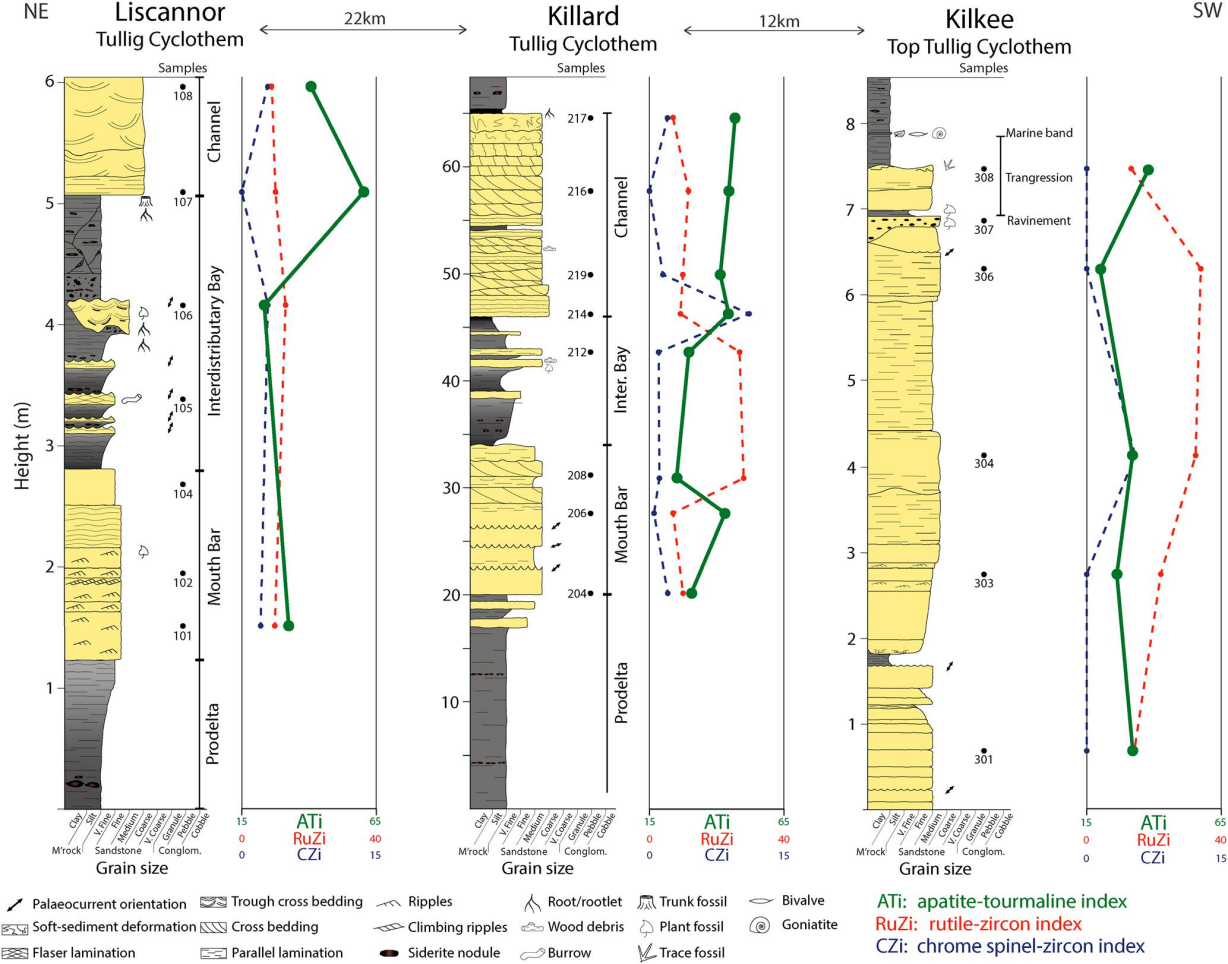
Sedimentary logs and three heavy mineral indices for each studied locality. Modified from Nauton‐Fourteu et al. (2020)

**Figure 6:**
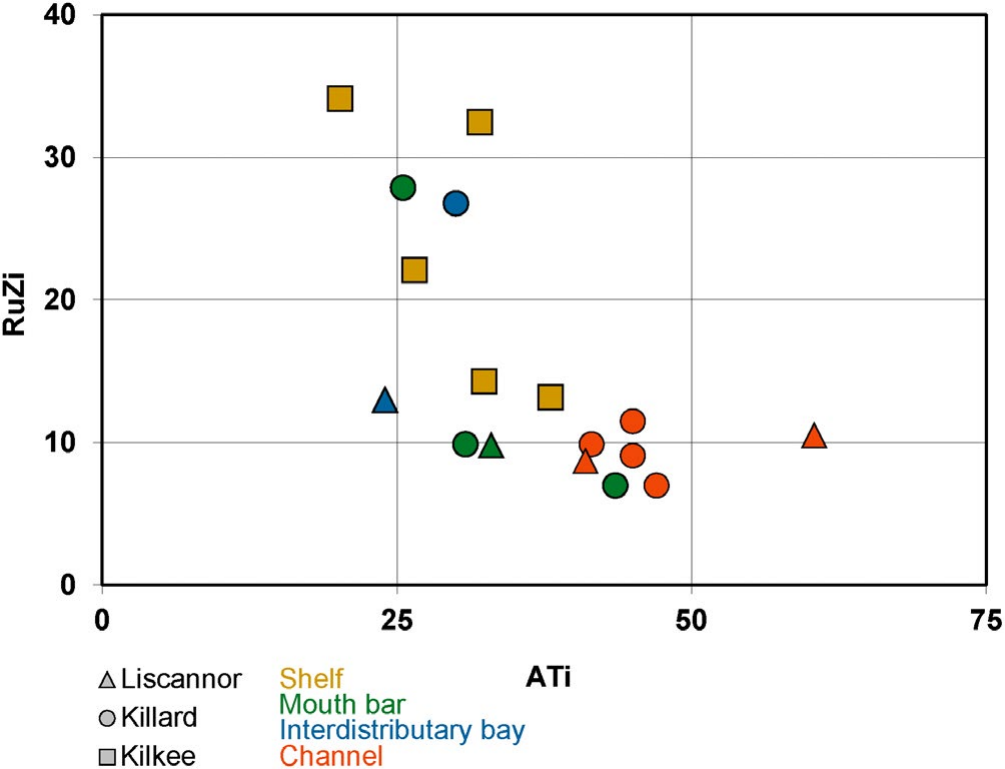
Binary plot of the rutile‐zircon index (RuZi) and the apatite–tourmaline index (ATi) from samples from three localities and four different facies

Heavy mineral indices (ATi, RuZi and CZi) have been plotted alongside the sedimentary logs for correlation with facies and in a RuZi‐ATi binary plot (Figures 5 and 6). Comparing results from the prograding delta observed at Liscannor and Killard, ATi values show lower values in mouth bar and interdistributary bay deposits than in the channelised Tullig Sandstone, except for one mouth bar sample at Killard. In Liscannor, RuZi and CZi values are relatively low and do not vary consistently with sedimentary facies. In Killard, RuZi values seem to vary, essentially ‘mirroring’ the pattern seen in the ATi, and the CZi values seem similar to Liscannor except for one sample at the base of the channel sandstone displaying a higher value. In Kilkee, no significant variations in heavy mineral indices are observed. ATi values are relatively low, and RuZi generally displays higher values than in Liscannor and Killard (13–34). The CZi is relatively low throughout the section.

## 5 DISCUSSION

### 5.1 Provenance indications from heavy mineral assemblages

The high ZTR indices observed throughout the quartz‐rich samples have been previously linked with a potential recycling of Old Red Sandstone from sedimentary basins to the south of the Clare Basin (Nauton‐Fourteu et al., 2020). Samples can be ascribed to either low (*ca* 7–14) or high (*ca* 22–34) RuZi values (Figure 6). Variations of the provenance sensitive RuZi index are usually believed to represent variations in sources (Hurst & Morton, 2014; Morton, 2012); however, this is not recorded in zircon and apatite geochronology data, which remains consistent throughout the sampled section (Nauton‐Fourteu et al., 2020). Chrome spinel observed, even at low amounts throughout the outcrop, could suggest a very minor input from a mafic to ultra‐mafic source, with a potentially more important input for one sample at the base of the Tullig Sandstone at Killard. Such an ultra‐mafic source could be the supra‐subduction‐zone ophiolite resulting from the closure of the Iapetus Ocean, as reported in the South Mayo Trough (Mange et al., 2003). The very low amounts or total absence of garnet, epidote and chloritoid could be due to deep burial and dissolution during diagenesis as these minerals are sensitive to such processes (Morton & Hallsworth, 2007; Turner & Morton, 2007).

### 5.2 Heavy mineral variations and sediment pre‐depositional history

**Figure 7:**
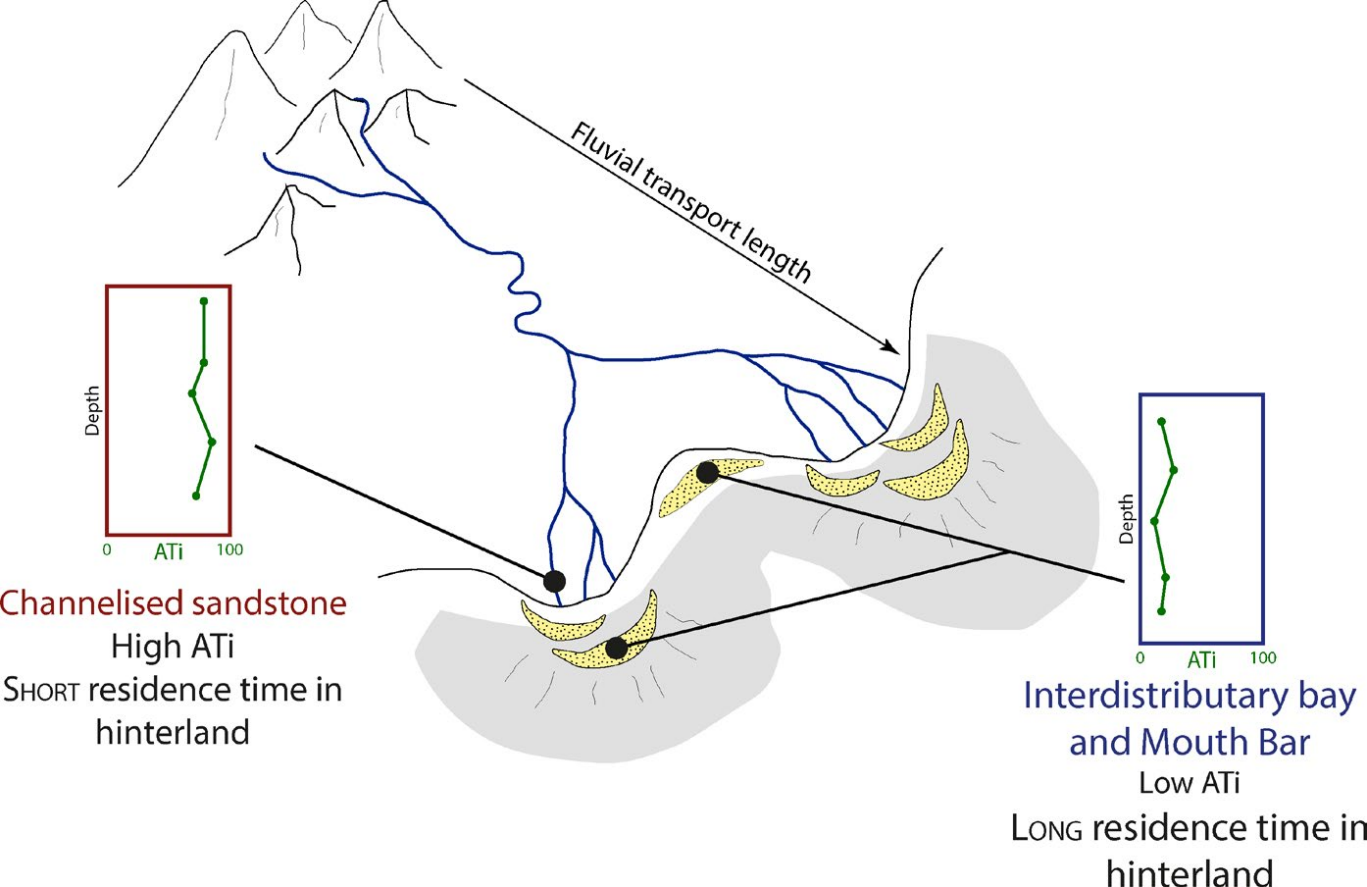
Schematic view of a potential interpretation. ATi: apatite–tourmaline index. Lower ATi values are observed in interdistributary bar and mouth bar deposits than in channelised sandstones, potentially highlighting a longer residence time in the system

Apatite is present throughout the 17 samples enabling the use of the ATi. Six of these samples have previously been analysed for zircon and apatite U‐Pb geochronology, illustrating no change of provenance across the different facies and the different locations (Nauton‐Fourteu et al., 2020). Hence, the relatively low ATi values in mouth bar and interdistributary bay deposits could be ascribed to a longer sediment residence time in the hinterland, including potential alluvial storage, with relative depletion of apatite compared to tourmaline through acidic weathering. In the channelised Tullig Sandstone, in contrast, ATi values are relatively higher, suggesting that the sediment potentially spent a shorter time in the hinterland, thus experiencing less chemical weathering (Figures 6 and 7). The one higher ATi value observed in the mouth bar in Killard could potentially be explained by a single event such as flooding, resulting in the flushing of sediments through the system and deposition in the basin. Such results agree with previous work where higher weathering intensities (low ATi values) were attributed to a longer residence time on the floodplain (Hurst & Morton, 2014; Morton et al., 2012).

Due to the negligible impact of hydrodynamic effects and chemical weathering on RuZi values (Morton & Hallsworth, 1994; 1999), variations observed in this index at Killard could be ascribed to changes in source rock. As noted above, a constant zircon and apatite geochronology provenance signal has been observed throughout the logged sections (Nauton‐Fourteu et al., 2020). The RuZi variations could thus be explained by changes in supply from a previously unidentified rutile‐rich, apatite/zircon poor source rock that is not captured or is significantly underrepresented in zircon and apatite geochronology. Zircon geochronology tends to be biased towards felsic igneous rocks, not detecting magma poor orogenies (Chew et al., 2020; O'Sullivan et al., 2016). Rutile is common in a range of rock‐types and especially abundant in high pressure, low temperature subduction metamorphic rocks (Chew et al., 2020; Meinhold, 2010; O'Sullivan et al., 2016). Previously reported apatite trace element data from sandstones from the Tullig Cyclothem highlight a source mainly from I‐type granitoids and mafic igneous rocks, with undateable grains derived from low‐grade and medium‐grade metamorphic rocks and some grains from high‐grade metamorphic rocks and S‐type granitoids (Nauton‐Fourteu et al., 2020). It is feasible, therefore, that the rutile (or at least a portion thereof) is ultimately being derived from the same or similar sources to that which supplies the undateable apatite. Nauton‐Fourteu et al. (2020) concluded that a portion of the apatite in the Tullig Cyclothem is ultimately derived from northern sources, but recycled into the Clare Basin through the Old Red Sandstone. The same could be the case for rutile, which may ultimately be derived from metamorphic rocks associated with the Caledonian Orogenic Cycle, potentially those associated with the subduction of Iapetean oceanic crust. These types of sources could also supply chrome spinel and explain the varying CZi values.

The RuZi‐ATi binary plot allows the two groups of samples to be distinguished, with five samples displaying high RuZi and 12 samples with low RuZi values. These data could illustrate that five samples have a higher contribution of the rutile‐rich source than the rest of the samples. Three of these five samples are from the shelf sandstones at Kilkee, one is from the mouth bar at Killard, and one is from the interdistributary bay at Killard (Figures 5 and 6). Additionally, channelised sandstones all display low RuZi values (Figures 5 and 6), potentially indicating that contribution from the rutile‐rich source is achieved through mixing of multiple sources on the shelf rather than from the point sources represented in and by the channelized sandstones. Furthermore, heavy mineral indices patterns in the Kilkee section (Figures 5 and 6) strongly contrast with those from Killard and Liscannor, and the limited variations in the respective indices could also highlight a potential homogenisation of sediment on the shelf through mixing by the action of waves, longshore drift and reworking associated with the steady rise of sea level at this specific time.

### 5.3 Heavy mineral indices as palaeoweathering and alluvial storage indicators

In this study, the different deltaic facies appear to manifest fluctuations in chemical weathering linked with variations in sediment residence time in the hinterland. This understanding suggests that palaeoweathering studies based on the modification of sediment composition should consider sedimentological facies and the sediment provenance history for accurate interpretation. Such variations in chemical weathering intensities can be observed within a single deltaic highstand system tract, and not only between highstand and lowstand conditions (Hurst & Morton, 2014; Morton et al., 2012). The ATi, in contrast to bulk rock geochemical proxies, allows for palaeoweathering information based on the dissolution of a chemically weathering‐sensitive single grain—apatite, over a more robust grain—tourmaline. Moreover, apatite can be dated using U‐Pb geochronology (Chew & Donelick, 2012) and trace elements can be used to further pinpoint sources (O'Sullivan et al., 2018; 2020). Apatite provenance within a sample can thus be well constrained and the identification of variations in sources from sample to sample can be assessed.

Sedimentary processes potentially obscuring the signal need to be considered in such a case study. As previously mentioned, burial diagenesis should not have an impact on the heavy mineral indices studied here as apatite and tourmaline are both resistant to deep burial diagenesis (Morton & Hallsworth, 1999; 2007). Recycling, however, has been identified and constrained in these samples from the Tullig Cyclothem of the Clare Basin (Nauton‐Fourteu et al., 2020). This previous multi‐proxy provenance approach identified a polycyclic origin for some of the detritus, with a potential recycling of apatite from the Galway and Donegal granites through the Devonian Old Red Sandstone south of the Clare Basin. In this study of polycyclic sediments that have experienced multiple weathering phases (Devonian and mid‐Carboniferous), the palaeoweathering signal observed within these sandstones seems to record the final transport phase, as the ATi varies with facies.

## 6 CONCLUSIONS

Heavy mineral indices, and more specifically the ATi, informs on pre‐depositional sediment history of Carboniferous deltaic sandstones. Different facies display variations in indices with no major change in provenance, hence these record the degree of chemical weathering that occurred in the hinterland, prior to deposition. Relatively lower ATi values observed in mouth bars and interdistributary bay sandstones potentially indicate more intense chemical weathering, which can be ascribed to longer residence times in the hinterland, than fluvial sediments which display higher ATi values. The provenance of the studied samples has been previously constrained, allowing for a detailed investigation of their heavy mineral indices. These results show that the ATi has potential as a palaeoweathering indicator even if the sandstones have had a complex multi‐cycle sedimentary origin. The link between relative ATi values and deltaic facies type demonstrates that these signals can potentially be used to interpret depositional environments in core samples, for which sedimentological information could be sparse. The RuZi variations observed in this study can be explained by variable supply from a source that is relatively poor in zircon and apatite (or in which any apatite is undateable), such that its signal is undetected in the zircon and apatite geochronological data. Such potential source rocks could also be the source supplying chrome spinel and be incorporated to the sediment composition through mixing on the shelf. Dating of rutile would potentially help better understand the provenance signal and the RuZi in these sandstones. This study highlights the importance of utilising a multi‐proxy provenance approach in order to understand more fully the sedimentary history of a target sedimentary rock or succession. A key aspect of this is the importance of constraining the sedimentological nature of each sample.

## Supporting information

Table S1

## Data Availability

The data that support the findings of this study are provided in Table S1.
